# Functional analysis of an essential Ran-binding protein gene, *CpRbp1*, from the chestnut blight fungus *Cryphonectria parasitica* using heterokaryon rescue

**DOI:** 10.1038/s41598-020-65036-7

**Published:** 2020-05-15

**Authors:** Yo-Han Ko, So-Yeon Choi, Kum-Kang So, Jung-Mi Kim, Jeesun Chun, Dae-Hyuk Kim

**Affiliations:** 1 Department of Molecular Biology, Department of Bioactive Material Sciences, Institute for Molecular Biology and Genetics, Jeonbuk National University, Jeonju, Chonbuk Korea; 2 Department of Bio-Environmental Chemistry, Wonkwang University, Iksan, Chonbuk Korea; 3 Department of Biological Sciences, Mississippi State University, Mississippi State, Mississippi USA

**Keywords:** Fungal biology, Fungal genetics

## Abstract

A Ran binding protein (RanBP) homolog, *CpRbp1*, from *Cryphonectria parasitica*, has been identified as a protein that is affected by hypovirus infection or tannic acid supplementation. In this study, functional analyses of *CpRbp1* were performed by constructing a knockout mutant and analyzing the resulting heterokaryon. Transformation-mediated gene replacement resulted in two putative *CpRbp1*-null mutants and genotype analyses identified these two mutants as heterokaryotic transformants consisting of two types of nuclei, one with the wild-type *CpRbp1* allele and another with the *CpRbp1*-null mutant allele. Although stable mycelial growth of the heterokaryotic transformant was observed on selective medium containing hygromycin B, neither germination nor growth of the resulting conidia, which were single-cell monokaryotic progeny, was observed on the medium. *In trans* complementation of heterokaryons using a full-length wild-type allele of the *CpRbp1* gene resulted in complemented transformants. These transformants sporulated single-cell monokaryotic conidia that were able to grow on media selective for replacing and/or complementing markers. These results clearly indicate that *CpRbp1* is an essential gene, and heterokaryons allowed the fungus to maintain lethal *CpRbp1*-null mutant nuclei. Moreover, *in trans* complementation of heterokaryons using chimeric structures of the *CpRbp1* gene allowed for analysis of its functional domains, which was previously hampered due to the lethality of the gene. In addition, *in trans* complementation using heterologous RanBP genes from *Aspergillus nidulans* was successful, suggesting that the function of RanBP is conserved during evolution. Furthermore, *in trans* complementation allowed for functional analyses of lethal orthologs. This study demonstrates that our fungal heterokaryon system can be applied effectively to determine whether a gene of interest is essential, perform functional analyses of a lethal gene, and analyze corresponding heterologous genes.

## Introduction

The chestnut blight fungus, *Cryphonectria parasitica* (Murrill) Barr, eradicated chestnut forests and orchards in North America at the beginning of the last century^1^. More interestingly, the presence of a single stranded RNA (ssRNA) hypovirus, Cryphonectria hypovirus 1 (CHV1), causes attenuation of fungal virulence, a phenomenon known as hypovirulence, and the symptoms associated with fungal infection^2–4^. As an ideal model system, *C. parasitica*-hypovirus interactions have been considered for investigating fungus-mycovirus interactions. This is possible due to the availability of various molecular tools that can be used for genetic manipulation of the fungus including gene replacement, heterokaryon rescue, and gene silencing. In addition, a highly annotated whole genome sequence database (http://genome.jgi-psf.org/Crypa2/Crypa2.home.html) is available and the application of an infectious cDNA clone of the hypovirus has been established to study the function of viral genes^5^.

Previous transcriptional and proteomic analyses in the presence of tannic acid (TA), which is found in high concentrations in chestnut bark and has a major role in defense against invading pathogens, led to the identification of proteins with differential expression^6^. One, Ran-binding protein 1 (RanBP1), drew our attention because it is an important regulator of the Ras-like nuclear small G protein (Ran), which is involved in nucleocytoplasmic transport and has never been analyzed in this fungus.

Ran is a highly conserved small GTP-binding protein that was initially proved as an essential element of the nucleocytoplasmic transport complex. It has since been implicated in many cellular functions, including various mitotic processes such as the regulation of DNA synthesis, centrosome formation, spindle assembly, nuclear envelope reformation^7^, and nuclear envelope structure in yeast^8,9^. It may also play a role in antiviral immunity in invertebrates^10^. Unlike other small G proteins, Ran localizes either in the cytosol or on the nuclear membrane and translocates between the cytosol and nucleus through nuclear complexes. The directionality of nucleocytoplasmic trafficking is maintained by a sharp gradient in the concentration of RanGTP between the nucleus and cytosol. Nucleotide binding to Ran is modulated by regulators such as guanine nucleotide exchange factor (RanGEF), Ran-binding proteins (RanBPs), and RanGTPase-activating protein (RanGAP). Thus, the gradient in the concentration of RanGTP is maintained by strict compartmentalization of these modulating proteins^11^.

RanBPs are characterized by a conserved Ran-binding domain (RBD) of ~140 amino acid residues, which is necessary and sufficient for nuclear transport of RanBPs and binding of RanGTP^12,13^. The variable and complex functions of Ran are largely dependent upon its binding partners^14^. However, RanBPs have some different functions independent of binding to Ran^15^. Although three RBD-containing proteins, Yrb1, Yrb2, and Nup2, have been well characterized in *Saccharomyces cerevisiae*^16–19^, studies on other filamentous fungi are very limited^20^.

Functional studies of these essential regulators in filamentous fungi have been challenging, mainly due to the difficulties obtaining appropriate mutants such as conditional mutants of typical temperature sensitivity^21,22^, replacing the native promoter with one that can be rapidly repressed^23,24^, and genome ploidy, as many important pathogenic fungi have an extended haploid phase. However, filamentous fungi may have the advantage of heterokaryosis, which is defined as the presence of two or even more genetically different nuclei in a common cytoplasm. The heterokaryon rescue technique allows analyses of whether a gene of interest is essential, as well as even further analyses of the terminal phenotype of the gene^25,26^. Stable heterokaryon formation has been observed in *C. parasitica*^27^, and a recent study demonstrated that heterokaryon formation of *C. parasitica* maintained mutant nuclei in which a gene of interest was lethal, providing the possibility of functional analyses of the corresponding lethal gene via complementation^28^. Therefore, although it is highly possible that RanBP1 of *C. parasitica* is important due to its participation in diverse cellular functions, we attempted to construct a RanBP1-null mutant to determine whether the function of RanBP1 is essential in *C. parasitica* and to obtain a heterokaryon for further functional analyses of the lethal RanBP1 gene. More than 100 small G proteins have been identified in eukaryotes from yeast to human. However, only limited studies have been conducted on small GTP-binding proteins. To the best of our knowledge, this is the first study on the function of RanBP1 in filamentous fungi using the heterokaryon rescue technique.

## Results

### Characterization of *CpRbp1* gene in response to TA and hypoviral infection

Among the 30 identified protein spots showing changes in accumulation by hypoviral infection or TA treatment, a protein spot tentatively identified as a homolog of RanBP was selected for further analyses^6^. The corresponding gene was identified by exploration of the genome sequence of *C. parasitica* (http://genome.jgi-psf.org/Crypa2/Crypa2.home.html). A 5,069-bp PCR amplicon containing the complete RanBP gene fragment was cloned, and sequence analyses of the cloned fragment demonstrated that the deduced amino acid sequences comprised the determined amino acid sequences of the gene. Based upon *in silico* analyses, a near full-length cDNA clone was obtained using the RT-PCR primer pair CpRbp1-cF1 and CpRbp1-cR1 at the positions of nucleotide (nt) -12 to 6 and 1202 to 1225 (1 is the first nucleotide of the start codon), respectively. Comparative analyses of the cDNA and genomic DNA sequences showed that the cloned gene comprised five exons, with four introns of 52 to 289, 443 to 564, 824 to 899, and 1029 to 1084 that were different from the prediction by ORF finder. The nucleotide sequence around the start codon matched to Kozak’s consensus sequence where the −3 position was the A in CAACATG. A putative polyadenylation signal sequence consisting of AAAAGA was found 10-bp downstream of the stop codon.

The deduced protein was composed of 238 amino acid residues, with an estimated molecular weight of 26.4 kDa. In addition, the protein was acidic, with a pI of 4.95 (GenBank No. MN053004). Sequence homology analysis using the deduced amino acids of the cloned gene revealed that it was similar to other fungal RanBPs from *Fusarium graminearum* (83.5% amino acid identity), *Metarhizium rileyi* (81.1%), *Colletotrichum simmondsii* (79.7%)*, C. nymphaeae* (79.7%), *Aspergillus nidulans* (79.3%), *C. fioriniae* (79.3%), and *Ustilaginoidea virens* (78.9%). Multiple alignment of seven closely related RanBPs showing an E value of ~0.0, as well as a founding member of the RanBPs, Yrb1 from *S. cerevisiae*, revealed that the protein product of the cloned gene had a multi-domain structure consisting of an N-terminal nuclear localization signal (NLS), a single canonical RBD in the central region, and an extended C-terminal coiled-coil region. Phylogenetic analyses indicated that the protein product of the cloned gene clustered together with fungal RanBPs, and those from *M. rileyi* and *U. virens* shared the most similar evolutionary lineage. The high bootstrap value of the phylogram suggested a genuine evolutionary relationship (Supplementary Fig. S1). Together with the presence of the hallmark RBD and significant homology to known fungal RanBPs, the cloned gene was referred as *CpRbp1* for *C. parasitica* RanBP1.

### Expression of *CpRbp1*

**Figure 1:**
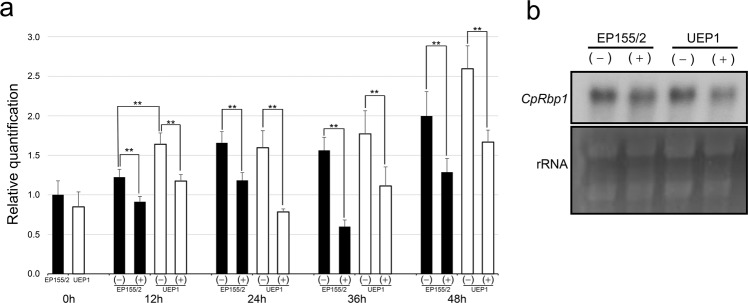
Expression analyses of *CpRbp1*. (**a**) qRT-PCR analyses of *CpRbp1*. Changes in expression of *CpRbp1* between the wild-type (EP155/2; indicated as solid bars) and hypovirulent (UEP1; indicated as open bars) strains relative to levels of glyceraldehyde-3-phosphate dehydrogenase (*gpd*) are indicated; (+) and (–) below the panel indicate with and without TA supplementation, respectively. Error bars indicate standard deviation based on three independent measurements. Numbers at the bottom indicate time after the transfer. ** indicates a significant change at *p* < 0.01. (**b**) Northern blot analyses of *CpRbp1* in response to hypovirus infection and TA supplementation. Total RNA was extracted from EP155/2 and UEP1 at 24 h after the transfer. Identification of the strains is shown at the top of the lanes; (+) and (–) above the panel indicate with and without TA supplementation, respectively. Equal loading of RNA samples is shown in the bottom panels by the ethidium bromide-stained gel (rRNA).

Since our previous proteomic analyses indicated that the protein product of the *CpRbp1* gene was upregulated by CHV1 infection or TA supplementation^6^, we examined the accumulation of gene transcripts under the corresponding conditions using Quantitative real-time reverse transcription PCR (qRT-PCR) (Fig. 1a). Contrary to the proteomic analyses, no significant changes in the accumulation of *CpRbp1* transcripts were observed due to CHV1 infection. However, the accumulation of *CpRbp1* transcripts was significantly changed in both wild-type and CHV1-infected UEP1 strains by TA supplementation. Interestingly, the opposite results were observed compared to the proteomic analyses, that is, the *CpRbp1* gene was downregulated instead of upregulatied in both wild-type and CHV1-infected UEP1 strains cultured on the TA-supplemented medium. Northern blot analyses of RNAs from both strains at 24 h after the transfer to either supplemented or non-supplemented media verified the qRT-PCR results, showing no changes due to CHV1 infection but downregulation of *CpRbp1* transcription by TA supplementation (Fig. 1b). These results clearly indicate that the accumulation of *CpRbp1* transcripts in *C. parasitica* was significantly affected in response to TA, a well-known and abundant host defense component in chestnut trees. However, as the transcriptional analyses were not consistent with the proteomic data, the regulation of *CpRbp1* expression appears to be far more complex than expected, occurring at the levels of transcription, post-transcription, and post-translation.

### Identification and characterization of putative *CpRbp1*-null mutants

**Figure 2:**
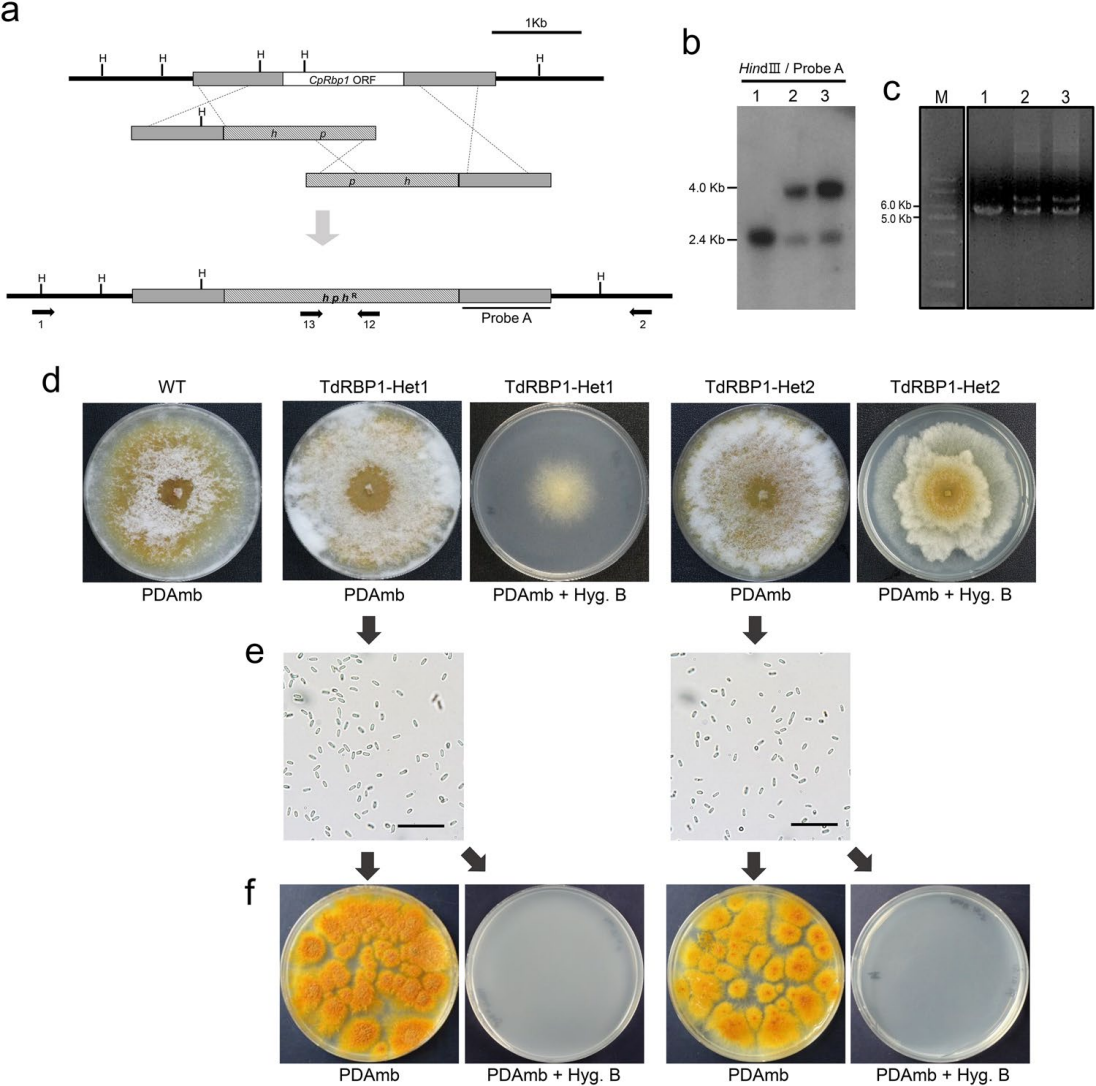
Identification of the *CpRbp1*-null mutant and segregation of conidia of the *CpRbp1*-null mutant strain. (**a**) Restriction map of the *CpRbp1* genomic region and the *CpRbp1*-null mutant with the desired replacement at *CpRbp1* are represented in the map together with the expected changes in the sizes of the restriction fragments. *hph*^R^, indicated by the dashed box, represents the hygromycin B resistance cassette. H represents restriction endonuclease *Hin*dIII. (**b**) Southern blot analyses of the wild-type EP155/2 strain (lane 1) and two heterokaryotic *CpRbp1*-null mutants (lanes 2 and 3). Enzyme/probe combination is indicated above the line, and the probe is indicated in the restriction map in the upper panel a. (**c**) PCR amplicons of the heterokaryotic *CpRbp1*-null mutants using the primer pair 1 & 2. Lanes 1, 2, and 3 indicate EP155/2 and heterokaryotic *CpRbp1*-null mutant strains, respectively. Lane M contains the 1.0 kb size marker. The relevant PCR primers for strain verification are denoted by arrows in the restriction map in the upper panel a. (**d**) The colony morphology after 10 days of culturing is shown. Even when the incubation period was extended, there were no signs of sporulation on PDAmb with hygromycin B. (**e**) Conidia harvested from the putative *CpRbp1*-null mutant strain grown on PDAmb for more than 14 days are shown. (**f**) 100 conidia were spread on PDAmb plates with or without hygromycin B. Strain identifications are provided above the picture. WT and TdRBP1-Het denote the EP155/2 and heterokaryotic *CpRbp1*-null mutant strains, respectively.

For functional analyses of the *CpRbp1* gene, we attempted to construct a *CpRbp1*-null mutant by integrative transformation-mediated gene replacement. A total of 92 stable transformants were screened by PCR as described previously, using two pairs of outer gene-specific primers and inner hygromycin B phosphotransferase gene cassette (*hph*) primers (Primers 1&12 and 2&13 in Fig. 2a) corresponding to nt −1,953 to −1,933 and 1,222 to 1,241, and nt 446 to 465 and 3,097 to 3,116, relative to the start codons of *CpRbp1* and *hph*, respectively. Two transformants showed 3,559-bp- and 3,521-bp PCR amplicons using 5′-proximal and 3′-proximal primer pairs, respectively, which corresponded to the expected sizes of the disrupted alleles of the *CpRbp1* gene (data not shown). In addition, these PCR amplicons were further confirmed by sequencing. However, Southern blot analyses of the two putative *CpRbp1*-null mutants using *Hin*dIII digestion and a probe prepared using the 1,004-bp PCR amplicon containing the 3′flanking region of the *CpRbp1* gene showed two hybridizing bands at 2.4 kb and 4.0 kb, corresponding to the wild-type and expected *CpRbp1*-null mutant alleles, respectively (Fig. 2b). PCR amplification using the outer gene-specific primers, located outside of the 5′ and 3′flanking regions of the replacement vector (Primers 1&2 in Fig. 2a), yielded two bands of 6,284 bp and 5,069 bp (Fig. 2c). Restriction enzyme analyses of PCR amplicons followed by sequencing analysis indicated that these PCR amplicons corresponded to the wild-type and *CpRbp1*-null mutant alleles. These results clearly indicate that the replacing *CpRbp1*-null mutant allele existed at the site of the *CpRbp1* gene, but the wild-type *CpRbp1* allele remained simultaneously in the putative *CpRbp1*-null transformants.

We, then, questioned whether the putative *CpRbp1*-null transformants were a simple mixture of wild-type and *CpRbp1*-null mutant strains or heterokaryons consisting of two different types of nuclei (i.e., one with the wild-type *CpRbp1* allele and the other with the *CpRbp1*-null mutant allele) in a single strain. Considering that our stable transformants were maintained by successive transfer of young hyphal tips to the selection media, it is highly unlikely that the putative *CpRbp1*-null transformants were simply a result of mixed cultures. Taking advantage of the fact that an asexual spore (conidium) of *C. parasitica* is a single cell containing a single nucleus^29^, a single-spore isolation is enough to resolve the mixed cultures and heterokaryon. Thus, a single-spore isolation, which can breakdown the heterokaryotic state during sporulation, can be applied as a common biological method to obtain a single cell monokaryotic progeny. Conidia from the putative transformants grown on the nonselective potato dextrose agar (PDA) plates supplemented with methionine and biotin (PDAmb) medium were harvested (Fig. 2d), microscopically inspected (Fig. 2e), diluted to the number of 10^2^ and 10^3^ per plate, and plated on the hygromycin B-containing selective PDAmb medium. No colonies were observed on the selective medium even after prolonged (>4 weeks) cultivation (Fig. 2f). However, when the same spore suspension was tested on PDAmb medium, numerous colonies, ranging from 20–30% of the expected number of colonies, were observed, and the resulting colonies were all hygromycin B-sensitive, that is, none were able to grow when transferred to the selective PDAmb medium containing hygromycin B. In addition, PCR amplification of the *CpRbp1* locus from the resulting colonies revealed the presence of the wild-type *CpRbp1* allele alone. These results suggest that the spore suspension consisted of a mixture of two types of spores: one that was viable but hygromycin B-sensitive, containing the wild-type *CpRbp1* allele, and another that was non-viable. Single-spore analyses of the putative *CpRbp1*-null transformants clearly excluded the possibility of a simple mixed culture of wild-type and *CpRbp1*-null mutant strains, and strongly suggested that the putative *CpRbp1*-null transformants were heterokaryon with two different nuclei containing either the wild-type or the null-type *CpRbp1* allele existing in a common cytoplasm.

To confirm that the non-viable spores contained the *CpRbp1*-null mutant allele, *in trans* complementation of the parental heterokaryotic strains were conducted with the wild-type allele of the *CpRbp1* and the geneticin-resistance selection marker. Complemented strains showing stable geneticin resistance were single-spored and subsequent colonies showing both hygromycin B- and geneticin-resistance were selected. PCR analyses of progeny showing hygromycin B- and geneticin resistance revealed the presence of both the wild-type and *CpRbp1*-null mutant alleles. However, colonies showing resistance to geneticin alone or susceptibility to both hygromycin B and geneticin revealed the presence of only the wild-type *CpRbp1* allele.

These results clearly indicate that the *CpRbp1* gene is essential, that is, its absence is lethal, and confirm that the parental mutant strains are heterokaryotic.

### Morphological and cultural characteristics of heterokaryons

**Figure 3:**
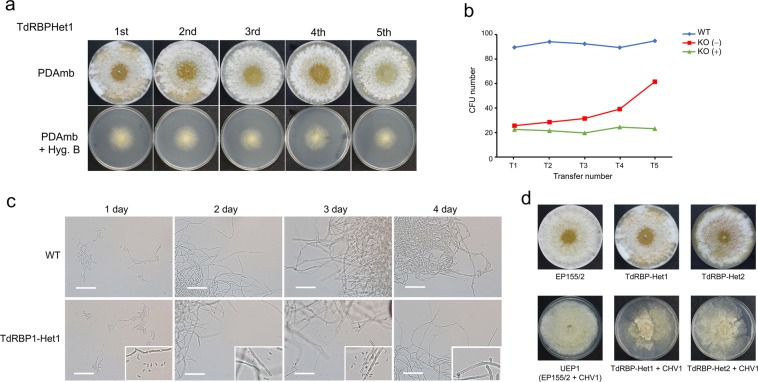
Characteristics of heterokaryon. (**a**) Colony morphology of the successive cultures of heterokaryon TdRBP1-Het1 on PDAmb and PDAmb supplemented with hygromycin B. (**b**) Estimated ratio of two conidia types from TdRBP1-Het1. TdRBP1-Het1 cultures were maintained by successive transfers on PDAmb plates and successive transfers on selective PDAmb plates containing hygromycin B for more than 3 months. Conidia were collected from the transferred plates every week up to 20 weeks (T1–T20), and colonies from every fifth passage were selected for conidia analysis. In all, 100 conidia per plate, as counted by a hemacytometer, were spread on PDAmb media, and then the number of CFUs was determined. The number of CFUs represents the number of conidia containing wild-type nuclei. The wild-type EP155/2 is included as a control for spore counts and viability of freshly harvested conidia. (**c**) Microscopic observation of temporal stages in conidial germination of the putative *CpRbp1*-null mutant. The incubation times are indicated above the panel. Appearance of gigantic spherical cells is indicated in inlets of the corresponding stages. The strains used to harvest conidia, indicated on the left, were the wild-type EP155/2 (WT) and heterokaryotic *CpRbp1*-null mutant (TdRBP1-Het1) strains. Scale bar = 50 μm. (**d**) Colony morphology of CHV1-infected heterokaryons. Strains are indicated at the bottom of the panel. Virus-free and virus-infected isogenic strains are indicated in the upper and lower panels, respectively.

The two heterokaryons were named TdRBP1-Het1 and TdRBP1-Het2. There were no differences in culture characteristics, and both strains were similar to the wild-type on nonselective medium (Fig. 2d); thus, TdRBP1-Het1 was selected for further analyses. Because the genotype frequency in a heterokaryon can vary depending on growth conditions^28,30^, we examined changes in the genotype frequency of heterokaryon progeny. The genotype frequency was estimated by measuring the ratio of colony-forming units (CFUs) from counted conidia of heterokaryon progeny that were successively transferred every fifth day for up to 20 transfers on selective and nonselective media (Fig. 3a). As shown in Fig. 3b, the heterokaryon TdRBP1-Het1 contained a lot more *CpRbp1*-null mutant nuclei than wild-type nuclei based on the fact that approximately 20% of observed conidia produced colonies at the beginning. When this heterokaryon was successively cultured on nonselective medium by passage every fifth day transfer, the number of CFUs, that is, the ratio of wild-type nuclei to *CpRbp1*-null mutant nuclei, gradually increased to 60% and remained thereafter. However, when TdRBP1-Het1 was cultured on selective medium, the wild-type frequency persisted at ~20%, suggesting that continuous selection pressure caused the fungus to maintain a high frequency of *CpRbp1*-null mutant nuclei for the hygromycin B resistance. The colony morphology of successively transferred heterokaryon progeny did not significantly change, and did not differ from that of the wild-type (Fig. 3a). These results indicate that the wild-type nuclei played a dominant role in determining the phenotype in heterokaryon, and that although the genotype frequency may change, balanced heterokaryons were maintained.

One of advantages using the heterokaryon rescue technique is the possiblity to determine the function of the null-allele to the terminal stage. As shown in Fig. 3c, no signs of early germination, such as swelling or germ tube formation, were observed from spores containing the mutant allele, suggesting that *CpRbp1* is crucial for the fundamental cellular function of this fungus and is required from the beginning of fungal metabolism.

We attempted to transfer CHV1 from the CHV1-infected hypovirulent UEP1 strain to the heterokaryon using hyphal fusion. After the successive transfer of hyphal tips on selective medium, the presence of CHV1 was confirmed and the resulting heterokaryon containing CHV1 was used for further analyses. The CHV1-containing heterokaryon displayed viral symptoms of reduced pigmentation and conidiation similar to UEP1 (Fig. 3d). Compared to the hypovirulent UEP1 strain, no significant changes in CHV1 accumulation were observed in the CHV1-infected heterokaryon.

### Functional analyses of the lethal *CpRbp1* gene using heterokaryons

**Figure 4:**
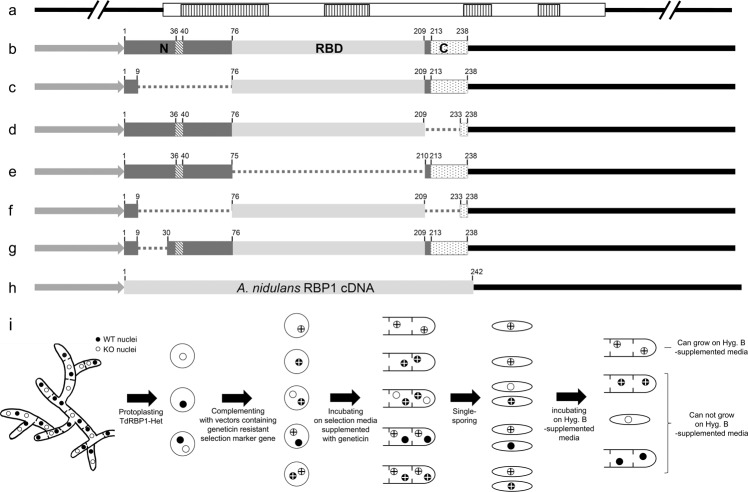
Schematic representation of the complementing vectors and expected outcomes of complementation. (**a**) Full-length genomic DNA of the *CpRbp1* gene was used as a control; vertical dashed lines in the genomic DNA represent introns. (**b–g**) A series of vector constructs containing various domains of CpRBP1 were constructed using cDNA of the *CpRbp1* gene and an expression cassette consisting of a strong constitutive cryparin promoter (p188) and *trpC* terminator. (**h**) A heterologous gene from *A*. *nidulans* was analyzed using the corresponding cDNA and the expression cassette. Functional domains including the N-terminal region, RBD, and C-terminal region are indicated as boxes marked with N, RBD, and C, respectively. The NLS within the N-terminal region and the coiled-coil domain within the C-terminal region are indicated by dashed and dotted boxes, respectively. The promoter and terminator are indicated by a grey arrow and solid line, respectively. Dotted lines represent deleted amino acid residues. Numbers indicate amino acid residues of CpRBP1. (**i**) Schematic diagram of possible outcomes of complementation with functional genes.

Functional analyses of lethal genes in filamentous fungi has been hampered due to limited sources of conditional mutants and tightly regulated gene expression systems. Since heterokaryon is a unique feature of filamentous fungi, allowing the maintenance of lethal genotypic nuclei due to the dominance of wild-type nuclei, we applied various chimeric structures of the *CpRbp1* gene to transform the heterokaryon and evaluate whether, and how efficiently, the mutated versions could complement the *CpRbp1*-null allele (Fig. 4a–h). Various complementing vectors with or without conserved representative domains such as the NLS, RBD, and coiled-coil region were used to transform heterokaryon and the resulting complementing progeny were analyzed (Table 1 and Fig. 4i). At least 20 geneticin-resistant transformants were selected for each construct. Single-spore analyses of each complementing transformant was conducted using PDAmb, PDAmb supplemented with hygromycin B, and PDAmb supplemented with geneticin. All but one mutated construct failed to complement the *CpRbp1*-null nuclei; conidia either did not grow on any media, or grew on PDAmb and/or PDAmb supplemented with geneticin but not on PDAmb supplemented with hygromycin B. In addition, no conidial progeny grew on both hygromycin B- and geneticin-supplemented media. These results clearly indicate that protoplasts for complementation were heterokaryotic, and the single-spored transformants showing geneticin resistance contained wild-type nuclei transformed by the corresponding complementing vectors. Thus, the presence of intact NLS, RBD, and coiled-coil regions was necessary for the function of the *CpRbp1* gene. However, the mutated *CpRbp1* gene construct missing only the 20 N-terminal amino acid residues prior to the NLS resulted in transformants whose conidia were able to grow on PDAmb supplemented with hygromycin B and PDAmb supplemented with both hygromycin B and geneticin. These results indicate that this *CpRbp1* variant was able to complement the *CpRbp1*-null nuclei, suggesting that the missing amino acid residues were not necessary for function of the *CpRbp1* gene. As a comparison, the RanBP1 ortholog from *A. nidulans* was found to complement *CpRbp1*-null nuclei, indicating the existence of functional conservation among fungal RanBP1s.

**Table 1:** Complementing count number of transformants and single-sporing result.

Vector constructs in Fig.4	Number of Transformant		CFU		
	PDAmb + geneticin	PDAmb + Hyg. B	PDAmb	PDAmb + geneticin	PDAmb + Hyg. B
a	41	30	29	25	18
b	45	34	32	27	19
c	48	34	34	26	0
d	43	32	30	23	0
e	49	36	35	25	0
f	52	39	35	26	0
g	42	32	28	25	20
h	52	40	39	35	27

## Discussion

Ran plays important roles in many fundamental cellular functions, which are achieved by various effectors. Among several RanBPs, RanBP1 functions as a crucial cofactor to stimulate RanGAP activities by dissociating RanGEF. Although significant progress has been achieved in clarifying the regulatory functions of RanBPs in animals and yeasts, very limited studies have been conducted on the function of RanBPs in filamentous fungi.

Analyses of the deduced amino acid sequence of a cloned CpRBP1 revealed the presence of conserved motifs within the RBD, including KXRAKLXRF, WKERGTGXXXXLXHK, and RXXMRRXKTLKXCANH (where X is any amino acid). However, RanBP1s from higher organisms show differences from their *S. cerevisiae* and *Schizosaccharomyces pombe* counterparts in both the sequence and the location of an extension outside of the conserved RBD. A classic monopartite NLS [K(K/R)X(K/R)] was found at residues 36 to 39, which is outside of the RBD and is also found in other filamentous fungi such as *A. nidulans*, *C. fioriniae*, *C. nymphaeae*, *C. simmondsii*, *F. graminearum*, *M. rileyi*, and *U. virens*. Interestingly, RanBP1s from *C. parasitica* and the evolutionarily related fungi mentioned above, excepting *A. nidulans*, showed amino acid extensions at their C-terminus, and CpRBP1 harbors a unique motif of a coiled-coil domain, which is known for a variety of important interactions^31^. Although the function of Ran may largely rely upon RanBP as its regulatory factor, RanBPs have been known to have some different functions depending on the organism, for example, RanBP1 is essential for viability in *S. cerevisiae* but not in mice^32^, and Nup2 is essential for viability in *A. nidulans* but not in *S. cerevisiae*^20^. In addition, different functions of RanBPs within the same organism have been demonstrated in *S. cerevisiae*, where Yrb1 was essential for cell viability, while Yrb2 and Nup2 had little or no apparent effects on growth. Moreover, new functions, independent of binding to Ran, extend the function of RanBP beyond its well-known role as a Ran effector^33–35^. Thus, differences in the functional domains of CpRBP1 strongly suggest that it may have several functions that differ from the founding member, Yrb1.

Sequence homology showed that the *CpRbp1* gene is highly similar to essential Yrb1 [E-value, 3e-75; amino acid identity, 145/238 (67%)], although not as similar as to RanBP genes from filamentous fungi, and function analyses of *CpRbp1* using heterokaryons proved that the *CpRbp1* gene is essential for cell viability. In addition, compared to other lethal genes exhibiting the terminal phenotypes of mutated genes^28,30^, no sign of initial germinating processes such as swollen spores was observed in conidia containing *CpRbp1*-null nuclei, suggesting that the *CpRbp1* gene product is involved in an initial fundamental cellular function that regulates many important processes for viability from the beginning.

Heterokaryosis, defined as the presence of two or more genetically different nuclei in a common cytoplasm, is a unique characteristic of a coenocytic fungus, in which cells contain multiple nuclei in a shared cytoplasm. The two most common ways of generating heterokaryon include mutation of any of the nuclei or mycelial fusion. Due to the coenocytic characteristics, mutated nuclei with recessive genotype can proliferate along with the wild-type nuclei. The heterokaryon characteristic of maintaining genetically different nuclei allows proliferation of nuclei with lethal genotypes, allowing further functional analyses. The heterokaryon rescue technique has been applied in *A. nidulans* to determine whether a gene of interest was essential for viability and to further analyze the functions of the essential gene by observing its terminal phenotypes^25,26^. However, this application is limited in other fungi and generalization of the technique to other fungal systems would require further studies of other organisms. Although the extent of heterokaryon of *C. parasitica* in natural environments is largely unknown, the presence of stable heterokaryon has been reported in nature^27^, and well-balanced maintenance of genetically engineered-lethal nuclei has been achieved^28,30^. Therefore, *C. parasitica* appears to be a good model fungus for studying essential genes based on the ease of detection and single-spore resolution, and more importantly, the balanced proliferation of genetically different nuclei, which is necessary for functional analyses using heterokaryon.

In eukaryotes, a larger number of small G proteins have been identified, and implicated in a wide range of cellular functions^14^. Numerous upstream regulators and downstream effectors of small G protein have gradually been characterized for their modes of actions^14^. Considering that signaling and biological functions of small G proteins with their effectors are conserved, the investigation of essential genes using fungal systems is intriguing. Not many studies on RanBP1 have been conducted in fungi other than yeast. Highly conserved motifs as well as unique features were identified by *in silico* analysis of the deduced amino acid sequence of the cloned *CpRbp1* gene. Although some RanBPs have already been identified as essential for viability, the occurrence of forced heterokaryon in *C. parasitica* facilitated our analyses of the function of a possible essential gene without the uncertainty of obtaining the corresponding mutant. Thus, the presence of heterokaryon and the following breakdown of the heterokaryotic state verified that the *CpRbp1* gene is essential. In addition, a complementation assay using heterokaryon confirmed that defined domain structures such as the N-terminal NLS, the RBD, and the C-terminal coiled-coil region of the *CpRbp1* gene were necessary for proper function of the *CpRbp1* gene. However, interestingly, although the coiled-coil region of the *CpRbp1* gene might be necessary for complementation of the *CpRbp1*-null nuclei, the ortholog from *A. nidulans*, which does not contain the coiled-coil region at the C-terminus, also complemented the *CpRbp1*-null nuclei. Although further studies are required to understand the structure and function of the coiled-coil in the C-terminal extension, homologous coiled-coil regions are important for adopting distinct conformations in the GTP- and GDP-bound states^36,37^. These results suggest that the coiled-coil region might be important for the intramolecular conformation, but not all RanBP1s require the presence of this structural motif. In addition, a fragment containing only the RBD of RanBP1 was enough to functionally complement a defective Yrb1 in yeast^38^. However our heterokaryon analyses indicated that other functional flanking regions were required. These results suggest that, although the sequence conservation is known among all RBDs from yeasts, plants, mammals, and other vertebrates, there are differences in the function of RanBP1 depending on the organism and the gene itself.

The *Rbp1* ortholog from *A. nidulans* complemented the deletion phenotype suggesting the functional conservation of RanBP1. This functional conservation of RanBP1 is promising and suggests that our heterokaryon analysis system of *C. parasitica* can be extended to analyze the biological functions of other heterologous genes as long as there are related fungal counterparts.

RanBP is involved in viral infection, including viral transport^39,40^ and host defense against viral infection^41,42^. Although CpRBP1 was affected by the presence of CHV1, no significant difference in viral symptoms or CHV1 accumulation was observed in CHV1-infected heterokaryon. These results suggest that the extent of wild-type nuclei in the heterokaryon is sufficient to reproduce viral symptoms in the fungus and support viral replication.

In this study, we investigated the biological functions of the RanBP1 gene of the chestnut blight fungus *C. parasitica*. Characterization of the putative *CpRbp1*-null mutants indicated that the mutants were heterokaryons consisting of two different types of nuclei carrying either the wild-type or the *CpRbp1*-null mutant allele in the common cytoplasm. In addition, single-spore resolution of the heterokaryotic *CpRbp1*-null mutants confirmed that the *CpRbp1* gene is essential. Moreover, complementation of heterokaryon using various constructs of the *CpRbp1* gene allowed us to determine that the conserved structural motifs including NLS, RBD, and coiled-coil were necessary for the function of the protein product of the *CpRbp1* gene. Functional conservation of the essential gene such as the *CpRbp1* gene further potentiate the application of heterokaryon to analyze the structure-function relationship of a lethal gene using this fungus.

## Methods

### Fungal strains and growth

*C. parasitica* strain EP155/2 (ATCC 38755) was maintained on PDAmb under constant low light at 25 °C (ref^43^.). For liquid culture of *C. parasitica*, EP complete medium was used^29^. The culture conditions and methods used to prepare the primary inoculum for liquid cultures have previously been described^43^. The mycelium was collected and lyophilized until use, as described previously^44^.

### Cloning and characterization of a RanBP1-like gene, *CpRbp1*

Proteomic analyses of previous studies revealed the amino acid sequences of selected protein spots. Search for the *C. parasitica* genome database (http://genome.jgi-psf.org/Crypa2/Crypa2.home.html) with the determined amino acid sequence identified the corresponding gene encoding an ortholog of the yeast RanBP, Yrb1. PCR amplification of genomic DNA using the primers CpRbp1-gF1 (forward) and CpRbp1-gR1 (reverse) was conducted to obtain a near full-length gene. The resulting 5,069-bp PCR amplicon was cloned and sequenced.

The cDNA clone of *CpRbp1* was obtained using PCR using reverse transcriptase (RT-PCR) with the primers CpRbp1-cF1 (forward) and CpRbp1-cR1 (reverse). The resulting 717-bp cDNA amplicon was cloned and sequenced.

The primers used to clone the *CpRbp1* gene and the various vector constructs for functional analyses of the gene are listed in Supplementary Table. 1.

### Southern and Northern blot analyses

Genomic DNA from *C. parasitica* was extracted according to a previously described method^45^. DNA (10 μg) was used to digest with the appropriate restriction enzymes, immobilized on a nylon membrane, and hybridized with radioactive-labeled probes.

RNA extraction from liquid and plate cultures and Northern blot analyses were conducted as previously described^6,46^. The level of *gpd* transcript was used as an internal control for gene expression of *C. parasitica*^47^.

### Construction of a replacement vector and fungal transformation

The gene replacement cassettes, which were applied to examine the biological effects of the *CpRbp1* gene, were constructed as described for split-marker deletion cassettes^48^. Two molecular DNA cassettes, each of which contained a part of the *hph* fused to either the 926-bp 5′ flanking region or 1,004-bp 3′ flanking region of *CpRbp1*, were prepared by overlap PCR^49^ as follows: A 954-bp PCR amplicon containing the 926-bp 5′ flanking region of *CpRbp1* was amplified using gene-specific primers Rbp1-F1 and Rbp1-R1 (Supplementary Table. 1). A 1,610-bp fragment of a selection marker gene containing the promoter and part of the N-terminus was amplified using primers Hph-F1 and Hph-R1 (Supplementary Table. 1). Fusion of these two PCR amplicons was conducted by overlap extension PCR with primers Rbp1-F1 and Hph-R1. A 1,024-bp PCR amplicon containing the 1,004-bp of 3′ flanking region and a 1,594-bp fragment of the selection marker gene containing the terminator and part of the C-terminus were amplified and fused using primer pairs Rbp1-F2/Rbp1-R2, Hph-F2/ Hph-R2, and Hph-F2/Rbp1-R2 (Supplementary Table. 1). The resulting molecular cassettes were then used simultaneously to transform protoplasts of *C. parasitica* EP155/2 strain.

Functional complementation of the *CpRbp1*-null mutant was performed using the wild-type allele. The complementing vector pCRbp1 was constructed by insertimg a 4,615-bp *Not*I fragment of pSilent-Dual1G (pSD1G) containing the geneticin resistance cassette^50^ into *Not*I-digested pRbp1 carrying a 5,559-bp fragment with the full-length *CpRbp1* gene. The resulting vector was then used to transform the putative *CpRbp1*-null mutant. For functional analyses of the *CpRbp1* gene, various chimeric structures of the *CpRbp1* gene were constructed using the cDNA clone of the *CpRbp1* gene and the constitutive expression cassette^51^. In addition, cDNAs of heterologous Rbp1 genes from *A. nidulans* (GenBank accession No XP_657688.1) was cloned and used for complementation.

Protoplast preparation and transformation were performed as previously described^43,45^. For *in trans* complementation, protoplasts of the putative *CpRbp1*-null mutant were obtained from young mycelial cultures grown in liquid medium inoculated with mycelial fragments instead of spores. Transformants were selected from agar plates supplemented with 150 μg/mL hygromycin B (Calbiochem, San Diego, CA, USA) or 150 μg/mL geneticin (Invitrogen, Carlsbad, CA) for replacement or complementation of *CpRbp1*, respectively. Cultures were passaged three to four times on selective media, and single-spores were isolated when possible, as previously described^30^. PCR and Southern blot analyses were conducted to confirm replacement and *in trans* complementation of the *CpRbp1* gene.

To analyze variation in the ratio of different types of nuclei in heterokaryons, the putative heterokaryotic transformant was cultured and successively transferred every fifth day for more than 3 months on nonselective and selective (containing hygromycin B) PDAmb media. Then, conidia were collected from plates representing every fifth transfer, and 100 conidia were spread on PDAmb plates with or without hygromycin B. The resulting CFUs were counted to determine the numbers of wild-type nuclei and *CpRbp1*-null mutant nuclei.

### Quantitative analyses of transcript accumulation by real-time RT-PCR

To examine the expression levels of target and internal control genes, quantitative RT-PCR (qRT-PCR) was performed using a GeneAmp 7500 sequence detection system (Applied Biosystems, Foster City, CA, USA) and a SYBR green mixture RT kit (Applied Biosystems) as previously described^52^. Analyses were conducted in triplicate for each transcript, from at least two independent RNA preparations of the same sample, using primers specific for g*pd* (RT-gpd-F1 and RT-gpd-R1) and *CpRbp1* (RT-Rbp1-F1 and RT-Rbp1-R1) (Supplementary Table. 1). Transcript abundance relative to the amount of *gpd* was analyzed using the 2^−ΔΔCT^ method^53^.

Accumulation of *CpRbp1* transcripts in the wild-type and hypovirulent UEP1 strains, with or without TA supplementation was analyzed by Student’s t-test at *p* < 0.01.

## Supplementary information

Supplementary information

## References

[1] Van Alfen NK (1982). Biology and potential for disease control of hypovirulence of *Endothia parasitica*. Ann. Rev. Phytopathol..

[2] Van Alfen NK, Jaynes RA, Anagnostakis SL, Day PR (1975). Chestnut blight: Biological control by transmissible hypovirulence in *Endothia parasitica*. Science.

[3] Anagnostakis SL (1982). Biological control of chestnut blight. Science.

[4] Nuss DL (1992). Biological control of chestnut blight: an example of virus-mediated attenuation of fungal pathogenesis. Microbiol. Rev..

[5] Choi GH, Nuss DL (1992). Hypovirulence of chestnut blight fungus conferred by an infectious viral cDNA. Science.

[6] Kim JM, Park JA, Kim DH (2012). Comparative proteomic analysis of chestnut blight fungus, *Cryphonectria parasitica*, under tannic-acid-inducing and hypovirus-regulating conditions. Can. J. Microbiol..

[7] Hetzer MW, Walther TC, Mattaj IW (2005). Pushing the envelope: structure, function, and dynamics of the nuclear periphery. Ann. Rev. Cell Dev. Biol..

[8] Demeter J, Morphew M, Sazer S (1995). A. mutation in the RCC1-related protein Pim1 results in nuclear envelope fragmentation in fission yeast. Proc. Natl. Acad. Sci. USA.

[9] Ryan KJ, McCaffery JM, Wente SR (2003). The Ran GTPase cycle is required for yeast nuclear pore complex assembly. J. Cell Biol..

[10] Liu H, Söderhäll K, Jiravanichpaisal P (2009). Antiviral immunity in crustaceans. Fish Shellfish Immunol..

[11] Gorlich D, Kutay U (1999). Transport between the cell nucleus and the cytoplasm. Annu. Rev. Cell Dev. Biol..

[12] Beddow AL, Richards SA, Orem NR, Macara IG (1995). The Ran/TC4 GTPase-binding domain: identification by expression cloning and characterization of a conserved sequence motif. Proc. Natl. Acad. Sci. USA.

[13] Künzler M, Gerstberger T, Stutz F, Bischoff FR, Hurt E (2000). Yeast Ran-binding protein 1 (Yrb1) shuttles between nucleus and cytoplasm and is exported from the nucleus via a CRM1 (XPO1)-dependent pathway. Mol. Cell. Biol..

[14] Takai Y, Takuya S, Takashi M (2001). Small GTP-binding proteins. Physiol. Rev..

[15] Dilworth DJ (2005). The mobile nucleoporin Nup2p and chromatin-bound Prp20p function in endogenous NPC-mediated transcriptional control. J. Cell Biol..

[16] Ouspenski II (1995). Ran-binding protein-1 is an essential component of the Ran/RCC1 molecular switch system in budding yeast. J. Biol. Chem..

[17] Dingwall C, Kandels-Lewis S, Seraphin B (1995). A family of Ran binding proteins that includes nucleoporins. Proc. Natl. Acad. Sci. USA.

[18] Hartmann E, Görlich D (1995). A Ran-binding motif in nuclear pore proteins. Trends Cell Biol..

[19] Koyama M, Shirai N, Matsuura Y (2014). Structural insights into how Yrb2p accelerates the assembly of the Xpo1p nuclear export complex. Cell Reports..

[20] Suresh S, Markossian S, Osmani AH, Osmani SA (2017). Mitotic nuclear pore complex segregation involves Nup2 in *Aspergillus nidulans*. J Cell Biol..

[21] Hartwell LH, Culotti J, Reid B (1970). Genetic control of the cell-division cycle in yeast. I. Detection of mutants. Proc. Natl. Acad. Sci. USA.

[22] Dohmen RJ, Wu P, Varshavsky A (1994). Heat-inducible degron: a method for constructing temperature-sensitive mutants. Science.

[23] Hughes TR, Evans SK, Weilbaecher RG, Lundblad V (2000). The Est3 protein is a subunit of yeast telomerase. Curr. Biol..

[24] Mnaimneh S (2004). Exploration of essential gene functions via titratable promoter alleles. Cell.

[25] Osmani SA, Engle DB, Doonan JH, Morris NR (1988). Spindle formation and chromatin condensation in cells blocked at interphase by mutation of a negative cell cycle control gene. Cell.

[26] Osmani AH, Oakley BR, Osmani SA (2006). Identification and analysis of essential *Aspergillus nidulans* genes using the heterokaryon rescue technique. Nat. Protoc..

[27] Anagnostakis SL (1981). A stable heterokaryon of *Endothia parasitica*. Mycologia.

[28] Ko YH, So KK, Kim JM, Kim DH (2016). Heterokaryon analysis of a Cdc48-like gene, *CpCdc48*, from the chestnut blight fungus *Cryphonectria parasitica* demonstrates it is essential for cell division and growth. Fungal Genet Biol..

[29] Puhalla JE, Anagnostakis SL (1971). Genetics and nutritional requirements of *Endothia parasitica*. Phytopathology.

[30] Kim MJ (2004). Deletion of a hypoviral-regulated *cppk1* gene in a chestnut blight fungus, *Cryphonectria parasitica*, results in microcolonies. Fungal Genet. Biol..

[31] Efimov VP, Morris NR (2000). The LIS1-related NUDF protein of *Aspergillus nidulans* interacts with the coiled-coil domain of the NUDE/RO11 protein. J. Cell Biol..

[32] Nagai M (2011). Mice lacking Ran binding protein 1 are viable and show male infertility. FEBS Lett..

[33] Kim SH, Arnold D, Lloyd A, Roux SJ (2001). Antisense expression of an Arabidopsis ran binding protein renders transgenic roots hypersensitive to auxin and alters auxin-induced root growth and development by arresting mitotic progress. Plant Cell.

[34] Tian B, Lin ZB, Ding Y, Ma QH (2006). Cloning and characterization of a cDNA encoding Ran binding protein from wheat. DNA Seq..

[35] Oliete-Calvo, P. *et al*. A role for Mog1 in H2Bub1 and H3K4me3 regulation affecting RNAPII transcription and mRNA export. *EMBO Rep*. **19**, 10.15252/embr.201845992 (2018). 10.15252/embr.201845992 PMC6216277 30249596

[36] Vetter IR, Nowak C, Nishimoto T, Kuhlmann J, Wittinghofer A (1999). Structure of a Ran-binding domain complexed with Ran bound to a GTP analogue: implications for nuclear transport. Nature.

[37] Nilsson J, Weis K, Kjems J (2002). The C-terminal extension of the small GTPase Ran is essential for defining the GDP-bound form. J. Mol. Biol..

[38] Petersen C, Orem N, Trueheart J, Thorner JW, Macara IG (2000). Random mutagenesis and functional analysis of the Ran-binding protein, RanBP1. J Biol Chem..

[39] Atkinson SC (2018). Recognition by host nuclear transport proteins drives disorder-to-order transition in Hendra virus V. Sci Rep..

[40] Yang YC (2015). RanBPM regulates Zta-mediated transcriptional activity in Epstein-Barr virus. J Gen Virol..

[41] Zhang Y (2018). RNA-binding protein YTHDF3 suppresses interferon-dependent antiviral responses by promoting FOXO3 translation. Proc. Natl. Acad. Sci. USA.

[42] Pan D, He N, Yang Z, Liu H, Xu X (2005). Differential gene expression profile in hepatopancreas of WSSV-resistant shrimp (*Penaeus japonicus*) by suppression subtractive hybridization. Dev. Comp. Immunol..

[43] Kim DH, Rigling D, Zhang L, Van Alfen NK (1995). A new extracellular *laccase* of *Cryphonectria parasitica* is revealed by deletion of *lac1*. Mol. Plant Microbe Interact..

[44] Powell WAJ, Van Alfen NK (1987). Two nonhomologus viruses of *Cryphonectria (Endothia) parasitica* reduce accumulation of specific virulence-associated polypeptides. J. Bacteriol..

[45] Churchill ACL, Ciuffetti LM, Hansen DR, Van Etten HD, Van Alfen NK (1990). Transformation of the fungal pathogen *Cryphonectria parasitica* with a variety of heterologous plasmids. Curr. Genet..

[46] Kim MJ (2002). Characterization of a fungal protein kinase from *Cryphonectria parasitica* and its transcriptional upregulation by hypovirus. Mol. Microbiol..

[47] Choi GH, Nuss DL (1990). Nucleotide sequence of the glyceraldehyde-3-phosphate dehydrogenase gene from *Cryphonectria parasitica*. Nucleic Acids Res..

[48] Goswami RS (2012). Targeted gene replacement in fungi using a split-marker approach. Methods Mol Biol..

[49] Sheppard SK (2005). Detection of secondary predation by PCR analyses of the gut contents of invertebrate generalist predators. Molecular Ecology.

[50] Nguyen QB (2008). Systematic functional analysis of calcium signalling proteins in the genome of the riceblast fungus, *Magnaporthe oryzae*, using a high-throughput RNA-silencing system. Mol. Microbiol..

[51] Kwon BR (2009). Assessment of the core cryparin promoter from *Cryphonectria parasitica* for heterologous expression in filamentous fungi. Appl. Microbiol. Biotechnol..

[52] Park JA, Kim JM, Park SM, Kim DH (2012). Characterization of CpSte11, MAPKKK gene of *Cryphonectria parasitica*, and initial evidence of its involvement in the pheromone response pathway. Mol. Plant Pathol..

[53] Livak KJ, Schmittgen TD (2001). Analysis of Relative Gene Expression Data Using Real-Time Quantitative PCR and the 2^−ΔΔC^_T_ Method. Methods.

