# Supplementary material for: Functional analysis of an essential Ran-binding protein gene, CpRbp1, from the chestnut blight fungus Cryphonectria parasitica using heterokaryon rescue
Source: Sci Rep. 2020 May 15;10:8111. doi: 10.1038/s41598-020-65036-7 (PMC7229160; doi:10.1038/s41598-020-65036-7)
Supplement: Supplementary file 1 — Supplementary information [file 41598_2020_65036_MOESM1_ESM.pdf]

Functional analysis of an essential Ran-binding protein gene, *CpRbp1*, from the chestnut blight fungus *Cryphonectria parasitica* using heterokaryon rescue

Yo-Han Ko[1], So-Yeon Choi[1], Kum-Kang So[1], Jung-Mi Kim[2], Jeesun Chun[1] and Dae-Hyuk Kim[1,*]

[1]Department of Molecular Biology, Department of Bioactive Material Sciences, Institute for Molecular Biology and Genetics, Jeonbuk National University, Jeonju, Chonbuk, Korea. [2]Department of Bio-Environmental Chemistry, Wonkwang University, Iksan, Chonbuk, Korea.

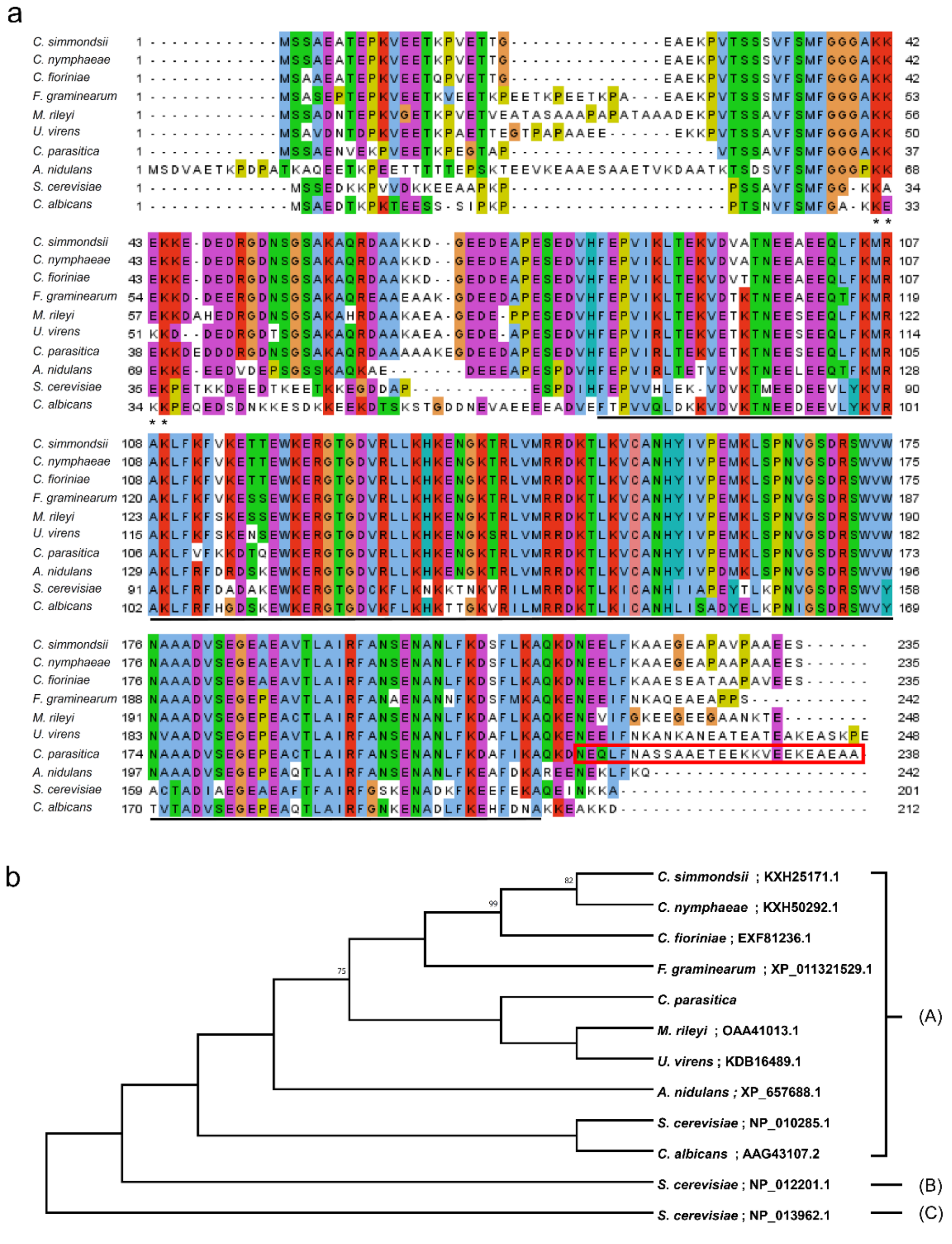

Supplementary Fig. S1 | Sequence similarity and phylogram. (**a**) Alignment of RanBPs with a canonical yeast Ran-binding protein 1 (YRB1) and other fungal RBPs. The amino acid sequence of the predicted CpRBP1 is highly conserved with other fungal RBPs and YRB1. Identical amino acids are highlighted in the same color. Dashes indicate gaps in the alignments. The RBD is underlined. The *in silico* predicted NLS sequence and coiled-coil region are indicated by asterisk and red box, respectively, in the amino acid sequence of

CpRBP1. (**b**) Phylogenetic tree of RBPs. A phylogenetic tree was generated using the amino acid sequences of the above RBPs and YRB1. An evolutionary matrix was generated by the Henikoff and Henikoff method[43], and the branching pattern was generated by the maximum likelihood method. Bootstrap values > 70% are shown. The GenBank accession numbers for the RBPs and YRB1 are listed next to each strain. (A) and (B) are fungal RBPs and YRB2, respectively. RanGAP (C) is included as an outgroup.

Supplementary Table 1 | List of PCR primer sequences

Primer Number	Primer Name	Primer Sequence (5'-3')	Use
1	CpRbp1-gF1	GTTCGGGGGCCTGCTCTCTG	Cloning of *CpRbp1* gene
2	CpRbp1-gR1	ATCCATGGTGGCTGGGCTGA	Cloning of *CpRbp1* gene
3	CpRbp1-cF1	CCTGTCGTCACCATGTCT	cDNA cloning of *CpRbp1*
4	CpRbp1-cR1	AATGTCCCAATTTTCTTTAAGCAG	cDNA cloning of *CpRbp1*
5	RT-gpd-F1	CTCTCAACACGGCAACTTCA	qRT-PCR
6	RT-gpd-R1	ACCAGTGGACTCGACAATG	qRT-PCR
7	RT-Rbp1-F1	AACCACTACATCGTGCCCGAG	qRT-PCR
8	RT-Rbp1-R1	GGTTGGCATTTTCGGAGTTGGC	qRT-PCR
9	Rbp1-F1	CGGCGTGCTTTGATATTCTG	Construction of *CpRbp1*-null mutant
10	Rbp1-R1	TCCTTCAATATCATCTTCTGTCGA CGTCACGACAGGTATATGGAGGG	Construction of *CpRbp1*-null mutant
11	HpH-F1	CCCTCCATATACCTGTCGTGACGT CGACAGAAGATGATATTGAAGGA	Construction of *CpRbp1*-null mutant
12	Hph-R1	ACCCGAAAACGCGTTTTATT	Construction of *CpRbp1*-null mutant
13	Hph-F2	ATTTCATATGCGCGATTGCT	Construction of *CpRbp1*-null mutant
14	Hph-R2	TTTAAGCAGCCTCAGCCTCCGTC GACGCTCTCCCTTATGC	Construction of *CpRbp1*-null mutant
15	Rbp1-F2	GCATAAGGGAGAGCGTCGACGG AGGCTGAGGCTGCTTAAA	Construction of *CpRbp1*-null mutant
16	Rbp1-R2	TCGGACCGATGGTCATTGTA	Construction of *CpRbp1*-null mutant

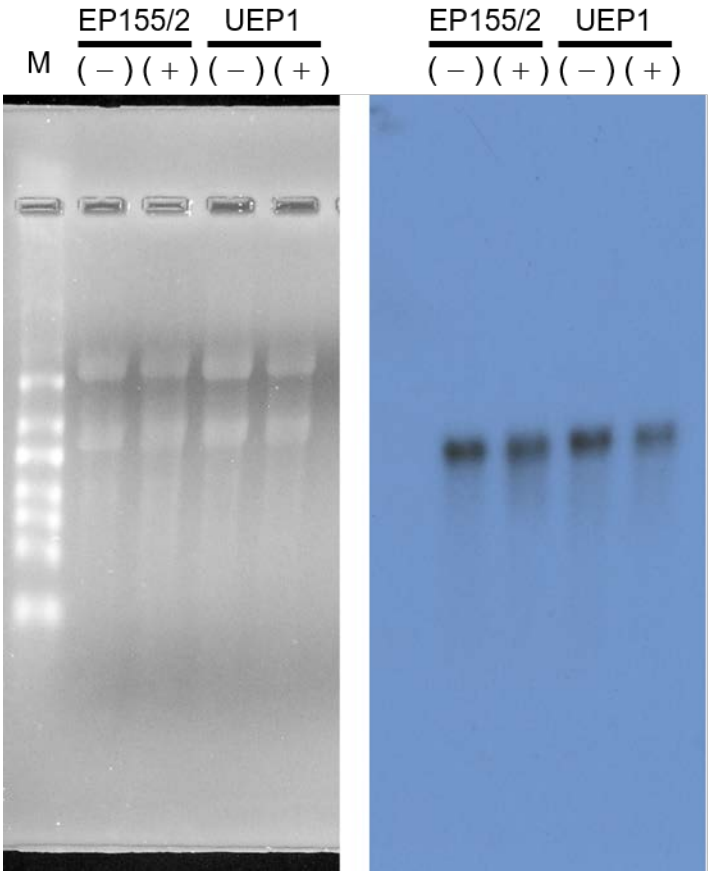

Full-length gel and blot of Fig. 1b

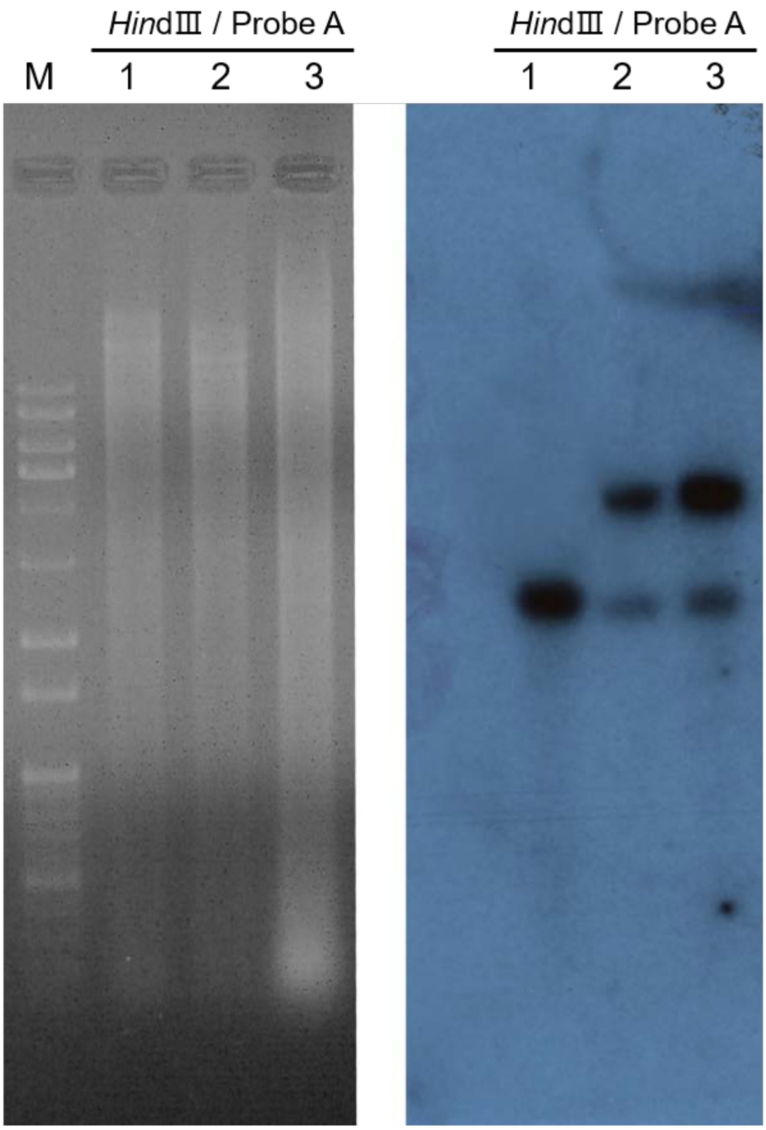

Full-length gel and blot of Fig. 2b

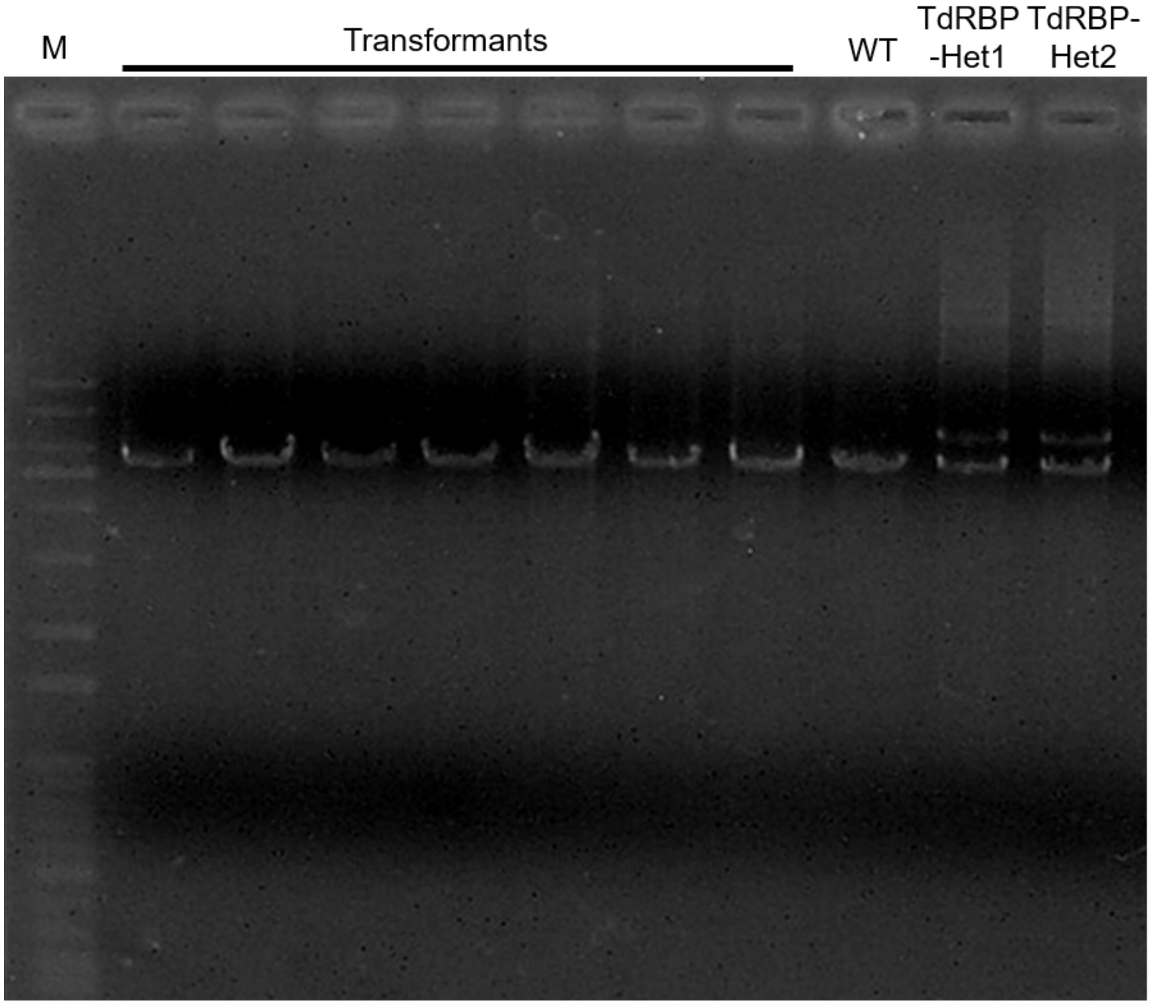

Full-length gel of Fig. 2c
